# A comparison of methods for calculating population exposure estimates of daily weather for health research

**DOI:** 10.1186/1476-072X-5-38

**Published:** 2006-09-13

**Authors:** Ivan Hanigan, Gillian Hall, Keith BG Dear

**Affiliations:** 1 School of Environmental Research, Charles Darwin University, Darwin, Northern Territory, 0909, Australia; 2 National Centre for Epidemiology and Population Health, The Australian National University, Canberra, Australian Capital Territory, 0200, Australia

## Abstract

**Background:**

To explain the possible effects of exposure to weather conditions on population health outcomes, weather data need to be calculated at a level in space and time that is appropriate for the health data. There are various ways of estimating exposure values from raw data collected at weather stations but the rationale for using one technique rather than another; the significance of the difference in the values obtained; and the effect these have on a research question are factors often not explicitly considered. In this study we compare different techniques for allocating weather data observations to small geographical areas and different options for weighting averages of these observations when calculating estimates of daily precipitation and temperature for Australian Postal Areas. Options that weight observations based on distance from population centroids and population size are more computationally intensive but give estimates that conceptually are more closely related to the experience of the population.

**Results:**

Options based on values derived from sites internal to postal areas, or from nearest neighbour sites – that is, using proximity polygons around weather stations intersected with postal areas – tended to include fewer stations' observations in their estimates, and missing values were common. Options based on observations from stations within 50 kilometres radius of centroids and weighting of data by distance from centroids gave more complete estimates. Using the geographic centroid of the postal area gave estimates that differed slightly from the population weighted centroids and the population weighted average of sub-unit estimates.

**Conclusion:**

To calculate daily weather exposure values for analysis of health outcome data for small areas, the use of data from weather stations internal to the area only, or from neighbouring weather stations (allocated by the use of proximity polygons), is too limited. The most appropriate method conceptually is the use of weather data from sites within 50 kilometres radius of the area weighted to population centres, but a simpler acceptable option is to weight to the geographic centroid.

## Background

A study of the possible effect of temperature and precipitation on gastroenteritis inspired an assessment of different methods for small area population exposure estimation techniques. The health data were obtained from a survey with respondents from Australia conducted between September 2001 and August 2002 [1]. In area-level analysis such as this the health outcome data and population exposure variables would ideally be available at the finest resolution of aggregation in space and time. The daily health outcome data were available for individuals and the postcode of their residence was recorded. The level of aggregation of weather observations for this analysis was also at the postcode level. The focus of this study was to therefore find the best method for representing the exposure of populations to daily weather in small geographic areas.

Within spatial units there are factors that may complicate the computation of exposure values. For instance there can potentially be large variation of temperature influenced by ground elevation. The design of the monitoring network can also have an impact. The density of weather recording stations is an important factor in computation of exposures, with more reliable estimates expected from areas that have many sites. The way the sites are spread throughout the area can also affect exposure estimates, such as whether they are evenly distributed or clustered together. An additional factor to consider when dealing with human health outcomes is the distribution of the population within the area, as our primary interest is in human exposure to environmental conditions.

The Australian Bureau of Statistics (ABS) does not publish census populations for postcodes, but instead for approximations termed Postal Areas (POA) [2]. Although there are inconsistencies when matching postcodes to POA [3], these were used in order to utilise information on the population distribution in the computation of exposure.

Population weighted exposure data are conceptually appealing as they more closely estimate the weather being experienced by the majority of the population. A complication is that some postcodes consist of multiple non-contiguous parts, or are large single-part postcodes with multiple population clusters. Therefore calculating an estimate for each sub-population separately gives better information from a population exposure perspective, although this is more computationally intensive.

Non-computationally intensive methods of calculating weather exposure estimates used by others have included taking the mean of all stations' observations within a geographic region, a method used by the Australian Bureau of Meteorology for precipitation since 1910 [4]. A similar method is to calculate the mean from the nearest neighbouring stations. This method has been used for a variety of purposes including rainfall [5,6]. A more sophisticated method is inverse distance weighted averages. This approach has been used in many area, point and gridding contexts [7,8].

An inverse distance weighted average is:

Q_j _= ∑W_ij_Z_i_/∑W_ij_

where Q_j _is the estimate of a day's weather for the jth spatial unit, Z_i _is the data value measured at the ith station, and the W_ij _are weights calculated as the reciprocal of the distance, or squared distance, between the jth spatial unit centroid and each of the stations in the neighbourhood. Stations outside the neighbourhood are given zero weight.

The inverse of the squared distance is most commonly used as the weight, however the inverse of the distance is also often used [9]. Using the inverse of the squared distance gives higher weight to closer observations. Note that the part of the spatial unit used to calculate distances from is a very important decision to be made. Some of the options available are: geographic centroid; population weighted centroid; the area boundary; sub-unit centroids; and sub-unit boundaries [10].

Other studies have compared the results from different methods for spatial interpolation of weather, focused on comparing the cells of gridded surfaces [11-14] or imputed data for stations with gaps [15]. The area estimates derived from different methods have been compared less often [16], and rarely in a population health context.

A recent study of health effects of air pollutants and weather in an Australian city estimated exposure at the aggregate level using the average of internal stations without assessing estimates weighted by distance or population [17]. This was noted as a possible limitation of the study design even though the authors considered that any measurement error would be "non-differential and produce conservative relative risks".

There has been much work in the air pollution research community investigating different methods of combining exposure data. Air pollution research that has addressed these issues includes those that compare modelled pollution (using dispersion models or geostatistical surface computation) and compared areal averages with those gained from simple averages of monitors [18-20], and others that use the distance from addresses or area centroids to monitors [21,22].

However in weather exposure studies often the rationale for using one technique rather than another is not explicitly considered and the differences in the values obtained by the different methods are generally unknown. Comparison between the results of the different estimates is required to ascertain the differential in particular contexts.

There has been a proliferation of approaches to the problems of spatial estimation of daily weather. Some of the methods are splining [23]; kriging and co-kriging [15]; gridded inverse distance weighting algorithms [4,11,24]; multiplicatively weighted proximity polygons [25]; artificial neural networks [26]; additive spatial regression models [27]; physically based numerical models of the three-dimensional atmospheric processes [28]; indirect methods such as radar [29]; and remote sensors mounted on satellites [30]. Some of these methods would enable the inclusion of relevant covariates such as elevation, wind speed and wind direction.

However there is no consensus about which is the best to use, some methods are computationally intensive and some commercially available options are expensive [31-33]. In addition, even if one of these were identified as a gold-standard to be used for creating gridded surfaces at each time point, it remains unclear whether it is worthwhile to undertake the extra computational burden needed to estimate population weighted exposure values. These could be based on fine resolution population distribution within spatial units, or on less computationally intensive approaches. It is not known which methods yield adequate weather estimates for health research. This paper addresses this important problem.

### Five methods for population exposure estimation

#### Option 1: average of internal or nearest neighbouring stations (using intersecting proximity polygons)

The first option used to estimate daily temperature and precipitation for POA was to calculate the average of internal stations, or the nearest neighbours if no internal stations exist. The first step in Option 1 was to identify stations in the POA boundaries. If there were no stations then the nearest neighbours were used. The "nearest neighbours" were found by the overlay and intersection of proximity polygons (also known as Thiessen or Voronoi polygons) with the POA boundary. In this approach each monitoring station is the focal point used to calculate the boundaries of a proximity polygon, whose area is defined so that all other points in it are nearest to the focal point than to any other focal point. The corresponding POA code is joined to each of the daily observations in a many-to-many relationship. Then the averages of each daily observation from the stations are calculated for each POA on each day.

#### Option 2: average of nearest neighbouring stations (using interesting proximity polygons)

The second option was to calculate the average of "nearest neighbours" regardless of their location inside or outside each POA boundary. Proximity polygons were used to allocate nearest neighbours as described in Option 1.

#### Option 3: geographic centroid inverse distance weighted average (using stations ≤ 50 km distant from centroid)

In the third option the distance between the geographic centroid and each station was used to calculate an inverse distance weighted average. The geographic centroid (also known as the mean centre) is the geographic centre of the boundary. The inverse distances from this centroid are used to weight the average of the station observations.

An arbitrary maximum distance of 50 km from the centroids of each spatial unit was used because it is likely that stations further away will not be similar to the area of interest [7]. The distance-weighting factor was also compared as the inverse of the distance (Option 3a) and the inverse of the squared distance (Option 3b).

#### Option 4: population weighted centroid inverse distance weighted average (using stations ≤ 50 km distant from centroid)

Option 4 used the distance between the POA population weighted centroid and the stations for an inverse distance weighted average. The population weighted centroid is calculated by subdividing the POA into its population census constituent sub-units (collector's districts) and calculating the centroids of these. The population-weighted centroid is found by weighting the average of the latitude and longitude coordinates of the sub-unit centroids by the populations of those sub-units. The choice of weights was also compared as the inverse of the distance (Option 4a) and the inverse of the squared distance (Option 4b).

#### Option 5: population weighted average of census collector's district distance weighted averages (using stations ≤ 50 km distant from centroid)

In the fifth option we calculated inverse distance weighted averages for each sub-unit geographic centroids (collector's districts) and then averaged these within POA using sub-unit populations as a weight. In this option each centroid had a weather estimate calculated for each day. Then the sizes of the populations are used to weight the contribution of these into each POA on each day. Option 5 differs from Option 4 in that it estimates the weather exposure for each sub-unit first and then gives a weighted summary of these for the POA. The choice of weights was also compared as the inverse of the distance (Option 5a) and the inverse of the squared distance (Option 5b).

### Examples

**Figure 1:**
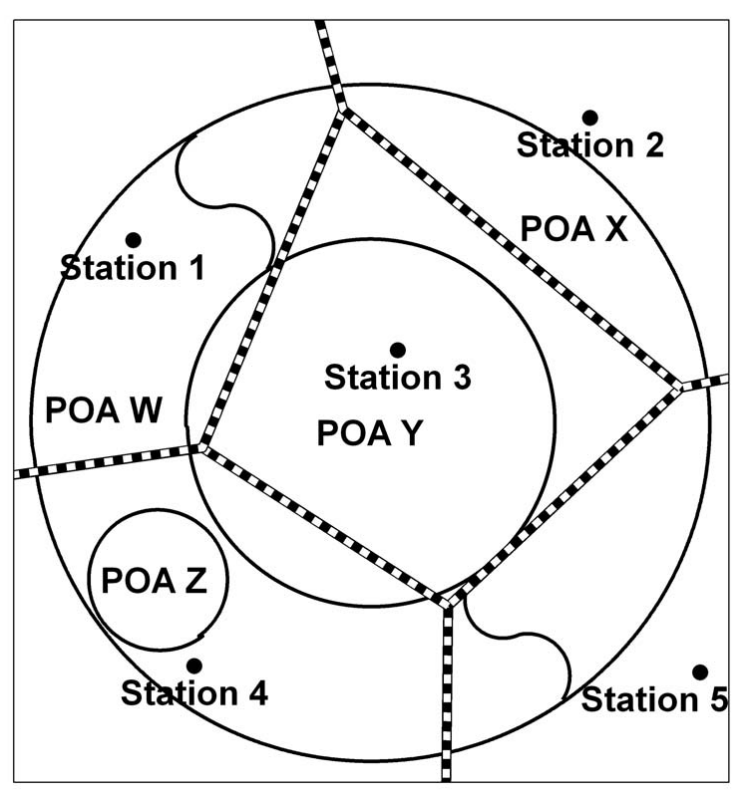
**Options 1 and 2 for calculating population exposure estimates of daily weather for areas**. Options 1 and 2 use the internal station and nearest neighbour by proximity polygon methods.

**Figure 2:**
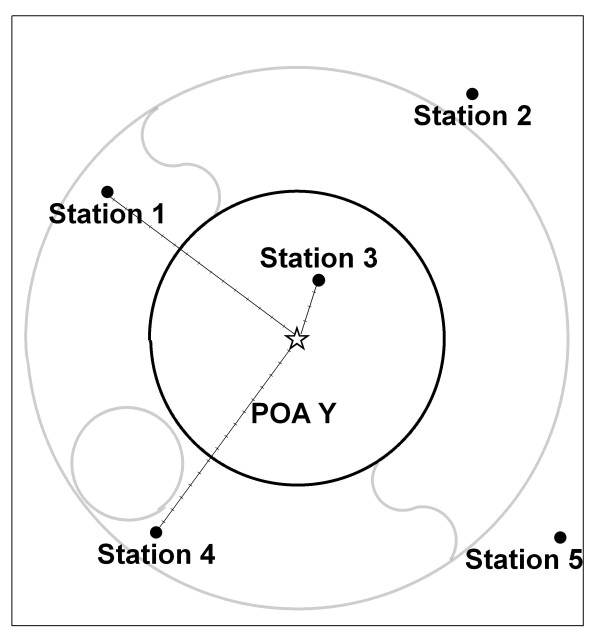
**Option 3 for calculating population exposure estimates of daily weather for areas**. Option 3 uses the inverse distance to geographic centroid.

**Figure 3:**
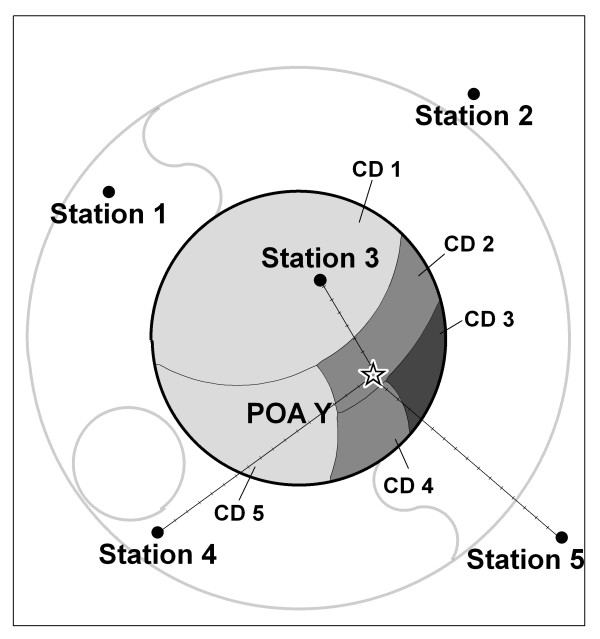
**Option 4 for calculating population exposure estimates of daily weather for areas**. Option 4 weights to the population weighted centroid.

**Figure 4:**
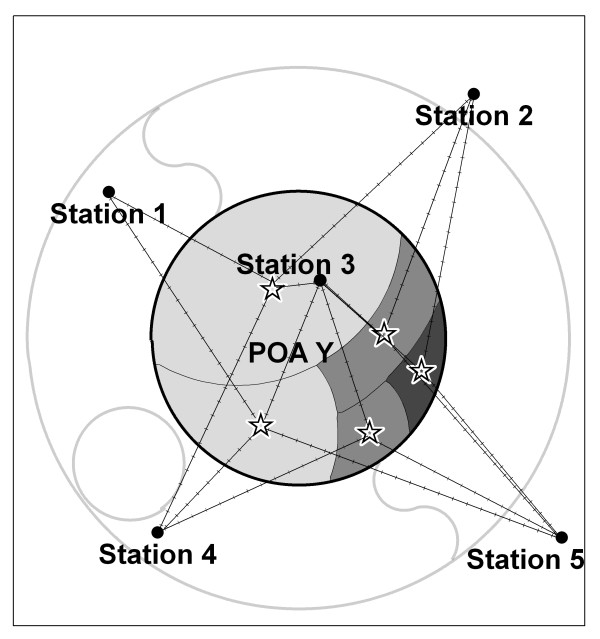
**Option 5 for calculating population exposure estimates of daily weather for areas**. Option 5 applies population weights to CD distance weighted averages.

**Figure 5:**
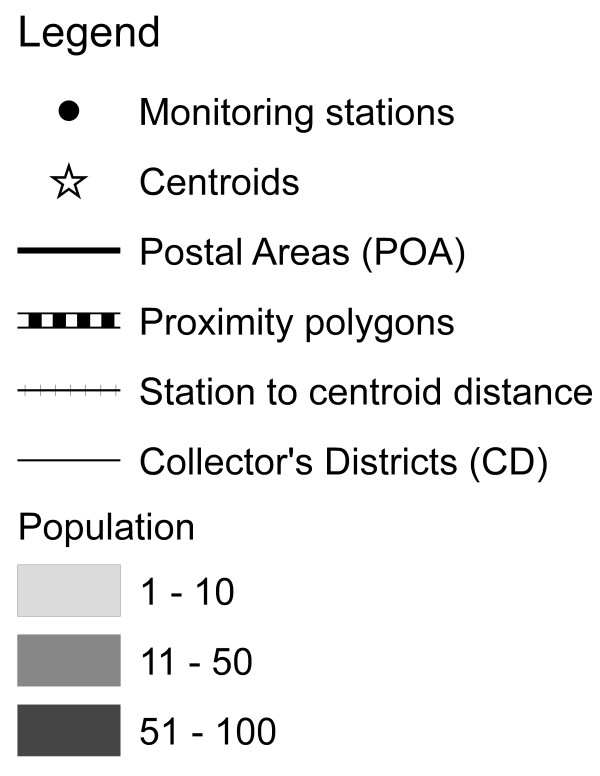
legend for symbols used in figures 1-4.

The options are shown as schematic diagrams in figures 1, 2, 3 and 4. Figure 5 shows the legend for the symbols used in these figures. For Options 1 and 2 the images are the same so we have displayed these two options together in figure 1. In Option 1 the areas with internal stations are assessed first. This would give POA Y the value of its internal station 3. POA W would be given an average of the two internal stations 1 and 4. Then the areas with no internal stations are assessed using the proximity polygon network represented by the thick dashed lines. POA X has four neighbouring stations 1, 2, 3 and 5. POA Z only has one overlapping proximity polygon indicating that the nearest neighbour is station 4.

In Option 2 the only differences are that now POA Y is given the average of the internal station 3 AND the nearest neighbouring stations 1 and 4. POA W now includes the neighbouring station 5 in the average of internal stations 1 and 4.

In Options 3–5 the process is only described for POA Y to avoid excessive detail. In figure 2, Option 3 is shown. The distances from the stations to the geographic centroid of POA Y (shown by the star) are calculated. The distances between the centroid and stations within the search radius are shown by the lines. The inverse distance weighted average will include stations 1, 3 and 4. The station 3 is so close to the centroid that the inverse distance weighted average will be dominated by this observation. This is especially the case using the weight calculated by the reciprocal of the squared distance.

Figure 3 shows that Option 4 uses a centroid weighted by the population of the sub-unit collector's districts (CD) to calculate the distances from the stations. For POA Y the centroid is pulled to the southeast because of the dominance of population in that direction. Distances are calculated from this centroid to the stations within the search radius which now includes the stations 3, 4 and 5.

In figure 4, Option 5 is shown. Here the distance from each sub-unit centroid to each station within the search radius is used to calculate a daily estimate. These are then weighted by the population and aggregated to give a POA level estimate.

We considered Option 5b the most conceptually appealing because it incorporates fine resolution population distribution patterns and is more sensitive to observations close to these sub-populations than the other options.

## Data

### Meteorological data

We obtained average daily temperature (the average of daily maximum and minimum temperature) in degrees Celsius and the daily precipitation in the 24 hours before 9 am in millimetres from the National Climate Centre of the Bureau of Meteorology Research Centre [34].

**Figure 6:**
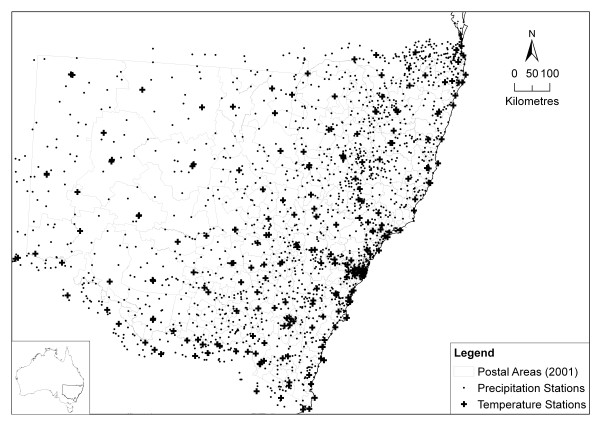
Map of Bureau of Meteorology monitoring stations overlayed on Postal Areas.

Weather data were obtained for 2,246 Bureau of Meteorology stations within 50 km of the NSW border (figure 6). Exposure estimates were calculated to correspond to the gastroenteritis survey respondent dates and postcode localities for the period August 2001 to December 2002 for 620 POA from NSW and the ACT. Not all stations in the relevant POAs logged observations in the period, nor do they all observe every parameter. The Meteorology Bureau receives data either electronically or manually on paper forms. These data may undergo initial error checking or subsequent error checking and each observation is given a quality rating. As a result of this error checking, and incorporation of additional historical data, the data may be modified. It is unlikely that there would be significant modifications made more than a few months after the date of observation [15]. 86% of the precipitation observations and over 99% of the temperature observations were considered acceptable by the Bureau. These were the only data used in this study. There were 1,816 stations with at least one good quality precipitation observation and 220 stations with at least one good quality temperature measurement during the period.

### Postcodes and postal areas

Inconsistencies between Australian postcode areas (for which health data are available) and Australian Bureau of Statistics POA boundaries (for which population data are available) are sometimes considerable [3,35]. Despite this we used the POA boundaries to enable the incorporation of fine resolution population data from the Australian Census [36]. The population data are based on the smaller CDs, which are then combined, by the Australian Bureau of Statistics, into the larger POA units in such a way as to align them as closely as possible to postcodes.

When a CD crosses more than one postcode, the decision rule for allocating it to a POA is the area that contains the majority of the population [37]. This is done in a subjective way using indicators such as how much of the area of the CD lies in each region and the distribution of land-use parcels [38].

A further complication is that some postcodes, and therefore some POA, comprise two or more separate land areas. In 2001 there were 72 such multipart POA in NSW and the ACT. The maximum distance between the geographic centroids of any two parts of the same POA was 350 km (POA 2831) and the mean of the split POAs centroid to centroid distance was 33 km. The maximum number of parts in any one POA was 16 (POA 2324). This is a common problem in coastal areas with many small islands allocated a single code, however these cases normally have small distances between parts. There are some inland POA with fewer numbers of parts but greater distances between these, due to the way Australia Post operates its delivery system.

## Results

### Summary of estimates from all five options

The time taken to calculate exposure estimates using Options 1 and 2 (2–3 hours per weather parameter on a desktop PC) was appreciably less than that required for Options 3 and 4 (around 8 hours per parameter). Option 5 required much more processing than the other options because each CD needed an inverse distance weighted weather estimate on each day (approximately 9,500 CD). This method was completed using a Structured Query Language server. Even using this more powerful computer, the time taken was approximately 8 hours per parameter.

The monitoring network is sparse in the west of the state and Options 1 and 2 suffer from a paucity of neighbourhood proximity polygon information. Of the 620 NSW and ACT POA, there were 375 that had internal precipitation stations and 130 with internal temperature stations.

Some POA are allocated only one station and when those stations had days with no observations this resulted in many days with missing data. The percentage of complete (and 90% complete) daily POA estimates are shown for each option in table 1. There are more gaps from the temperature observation network, which is sparser than the precipitation observations.

**Table 1:** Percentage of POAs with complete, and a majority, of weather estimates by option

	**Precipitation**		**Average temperature**	
**Option**	**100% days with estimates**	**>=90% days with estimates**	**100% days with estimates**	**>=90% days with estimates**

1	60%	95%	51%	94%
2	80%	98%	61%	96%
3	100%	100%	85%	95%
4	100%	100%	86%	96%
5	100%	100%	93%	98%

The problem stems from the fact that proximity polygon size is inversely related to the density of monitoring stations. In sparsely monitored regions the large size of polygons increases the probability that a POA will be allocated to only one monitoring station, causing gaps in the series on days when no weather information is available for that station. The example of POA Z in figure 1 shows that the estimate is determined by one station in Options 1 and 2. In contrast the distance-weighting scheme uses all stations within a given distance (50 km for this study) and thus incorporates more information.

The inverse distance weighting methods overcame this problem because it is more likely that there will be another station observing which could be used, and the information from these will be incorporated even if the nearest neighbour is not observing on a particular day. However this may cause some problems on days when there are only distant stations observing and these are given full weight because there are no close observations.

### Difference between the options

The difference between Option 5b and the daily estimates of each of the options was calculated.

Many of the precipitation estimates were zero due to the dry conditions in NSW and the ACT and consequently many of the differences between the precipitation estimates of the options were also zero. In Option 1 67% of estimates were zero, in Option 2 62% of estimates, Option 3a and 3b 36%, in Option 4a and 4b 36% and in Option 5a and 5b 33% of daily estimates were zero. To examine the rainfall differences between Option 5b and the comparative option, summary statistics of the precipitation differences were calculated for all estimated values where either Option 5b or the comparative option had a value greater than zero. The mean and median of the daily differences in Table 2 represent the bias of that option against Option 5b, after excluding those readings where either option estimated zero rainfall.

**Table 2:** Summary of daily differences between each option with Option 5b for temperature and precipitation

	**Mean**	**Standard Deviation**	**Median**	**Minimum**	**Maximum**	**25–75 percentile range**	**5–95 percentile range**
**Temperature (Celsius)**							
Difference(1-5b)	-0.01	0.66	0.00	-8.83	6.77	0.44	1.96
Difference(2-5b)	0.01	0.74	0.01	-8.83	6.43	0.53	2.32
Difference(3a-5b)	-0.06	0.46	-0.02	-6.61	6.14	0.31	1.22
Difference(3b-5b)	-0.02	0.37	0.00	-6.61	6.14	0.05	0.78
Difference(4a-5b)	-0.04	0.30	-0.02	-4.24	2.72	0.25	0.95
Difference(4b-5b)	0.00	0.16	0.00	-4.24	2.85	0.01	0.31
Difference(5a-5b)	-0.04	0.25	-0.01	-2.24	2.64	0.20	0.83
**Precipitation (millimetres)**							
Difference(1-5b)	0.08	2.57	0.01	-64.54	115.40	0.80	5.63
Difference(2-5b)	0.02	2.38	0.01	-102.90	115.40	0.66	5.63
Difference(3a-5b)	-0.02	1.78	0.01	-121.27	45.55	0.15	2.97
Difference(3b-5b)	-0.01	1.41	0.00	-128.70	103.17	0.02	1.22
Difference(4a-5b)	-0.02	1.36	0.01	-49.14	50.09	0.12	2.41
Difference(4b-5b)	0.00	0.65	0.00	-33.65	102.90	0.01	0.54
Difference(5a-5b)	-0.01	1.33	0.01	-49.31	21.41	0.12	2.31

In Option 1 the mean of the temperature differences is negative, implying that this option estimates lower temperatures on average than Option 5b. On the other hand the mean of Option 2 differences is positive implying that this option estimates higher temperatures on average. In the precipitation estimates for options 1 and 2 the mean is positive, implying higher rainfall estimates than Option 5b. The range and standard deviation for the differences for these options is large implying that the results are broadly inconsistent with the Option 5b estimates.

For the inverse distance weighted options (3, 4 and 5a) in both precipitation and temperature the mean difference shows that there is a tendency for Option 5b to have higher values with Option 4b the closest to 5b and 3a the most different. However, for precipitation the median differences are all positive or zero, suggesting that the mean is affected by some extreme values where rainfall estimates by Option 5b are considerably higher than the comparative option.

For temperature both the median and the mean are negative or zero for all options apart from Option 2, which implies that Option 5b consistently estimates higher temperatures.

**Figure 7:**
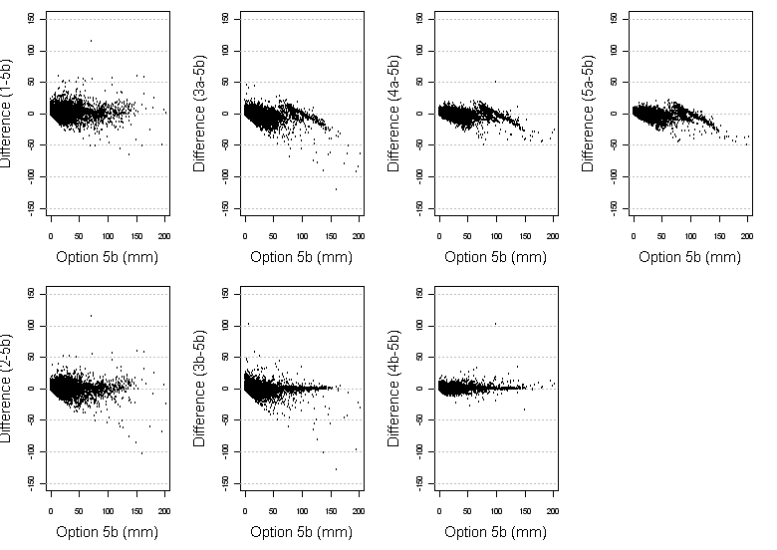
Scatter plots of the differences between precipitation estimates from each Option with Option 5b.

The scatter plots in figure 7 show the difference in precipitation for each of the options with Option 5b on the y-axis, and the magnitude of Option 5b on the x-axis. This shows that options 1 and 2 give very different estimates than Option 5b. In addition Option 5b gives markedly higher estimates than any of the non-squared weighted averages (3a, 4a and 5a) when considered with precipitation of greater magnitude. This probably represents the correlation between population density and rainfall, with more population clusters in areas where there is generally higher rainfall.

The differences between Option 5b rainfall and the inverse distance weighted options on the bottom row (3b and 4b) show that the difference generally does not vary when dealing with rainfall of greater magnitude (with a very few extreme exceptions such as a few precipitation differences greater than 100 mm). This implies that increasing the local weighting by squaring the distance changes estimates from Options 3 and 4.

**Figure 8:**
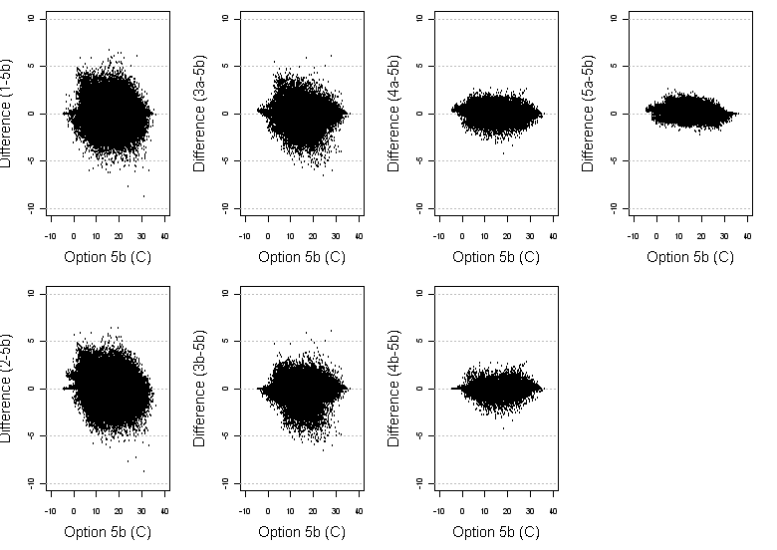
Scatter plots of the differences between temperature estimates from each Option with Option 5b.

The different temperature estimates are displayed in figure 8. The wider scatter in the first column show that the nearest neighbour methods perform poorly, with wide scatter. The scatter plots for Options 3a and 3b show that the geographic centroid gives quite different estimates, both greater and less than Option 5b, regardless of the weighting power. This is more evident in the mid-range of temperatures and the differences are reduced in the higher and lower temperature ranges. Option 4a and 4b give similar differences to Option 5a, and overall the differences for these are not great.

### Regional differences

**Figure 9:**
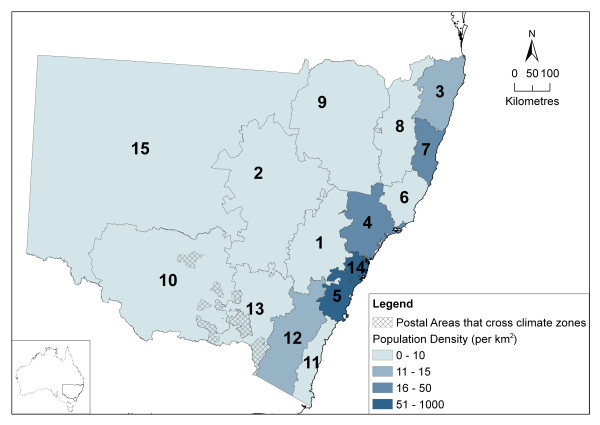
Postal Areas grouped into 15 climatic regions with population densities.

To see if differences between the options compared with Option 5b varied by region, we analysed the differences aggregated by 15 climatic zones. These zones were constructed by grouping POA based on the Bureau of Meteorology rainfall districts [39]. These climatic zones and their respective population densities are shown in figure 9. Two multipart POA that span the border of climatic zones (2652 and 2642), shown by the crosshatched areas were excluded because they cannot be considered part of a single climatic region.

**Figure 10:**
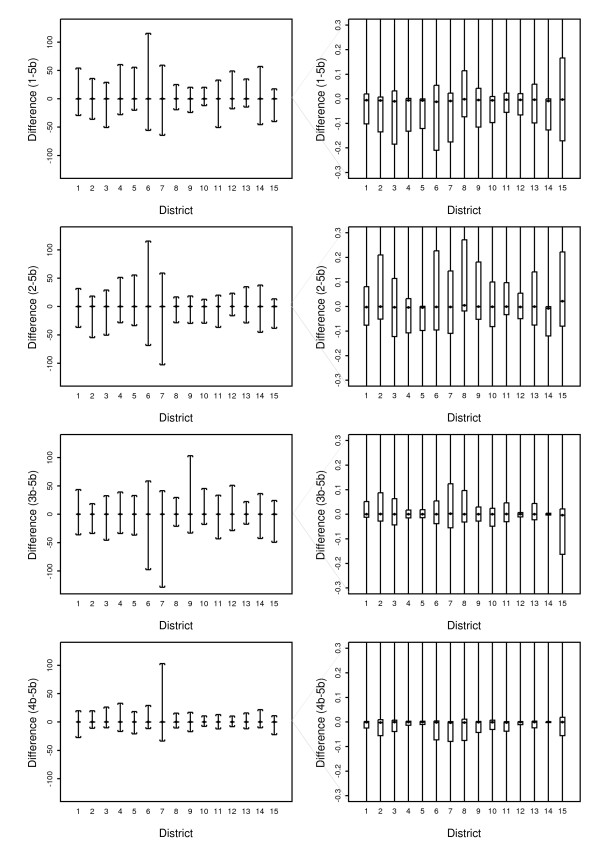
**Box plots of the precipitation differences for the 5 Options by climatic zone**. The left plot shows the range (whiskers) while the right hand plot shows the median, first quartile and the third quartile (box).

The daily precipitation differences (calculated only where either option is not zero) were grouped by region and displayed in the box and whisker plots in figure 10. These plots have been duplicated and the set on the left show the range of differences between the maximum and minimum (described by the top and bottom of the whiskers). The set on the right show in detail the range between -0.3 mm and 0.3 mm. The boxes contain all values between the first and third quartiles and this shows that 50% of the differences are very small in all districts. It also appears that the POA in districts 6, 7, 8 and 15 have larger rainfall differences from Option 5b (positive and negative) than other districts. The districts 6, 7 and 8 are in the higher rainfall zones of the NSW north coast where the spatial pattern of rainfall is usually highly localised. Region 15 is in the drier part of the state where there are larger POA.

**Figure 11:**
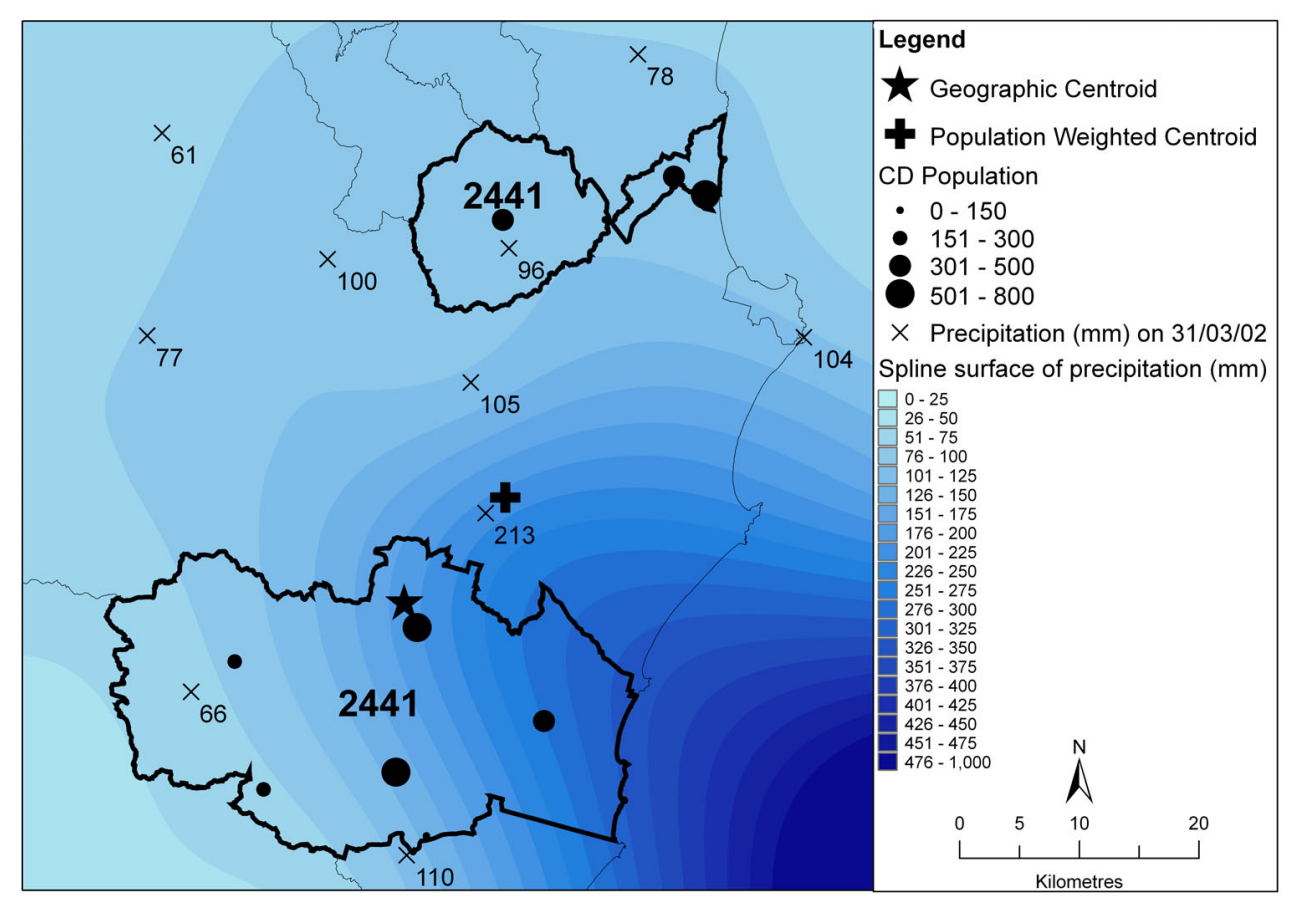
Example of precipitation over a multipart Postal Area giving different results for each option.

The influence of highly variable rainfall in a small area is demonstrated in figure 11 by an exemplar POA from the northern NSW coastal region (POA 2441). This POA happens to be split, and on the 31/3/2002 there was a storm that passed between two parts of the same POA that are about 20 km apart. The geographic centroid is shown by the star in the southern part of this POA. The parts have an equal sized population and so the population-weighted centroid (shown by the cross) falls equidistant between the parts (in multipart POA that have populations in each of the parts, the centroid will often fall outside the boundaries of the parts). The CD centroids are represented by the black circles, whose sizes are proportional to their populations. Option 1 had an estimate of 81 mm; Option 2 was 110 mm; Option 3a was 105 mm; Option 3b was 133 mm; Option 4a was 150 mm; Option 4b was 202 mm; Option 5a was 91 mm and Option 5b was 99 mm. Option 4 (the distance to population weighted centroid) was very different to Option 5 (population weighted average of distance to CD centroid weighted averages).

**Figure 12:**
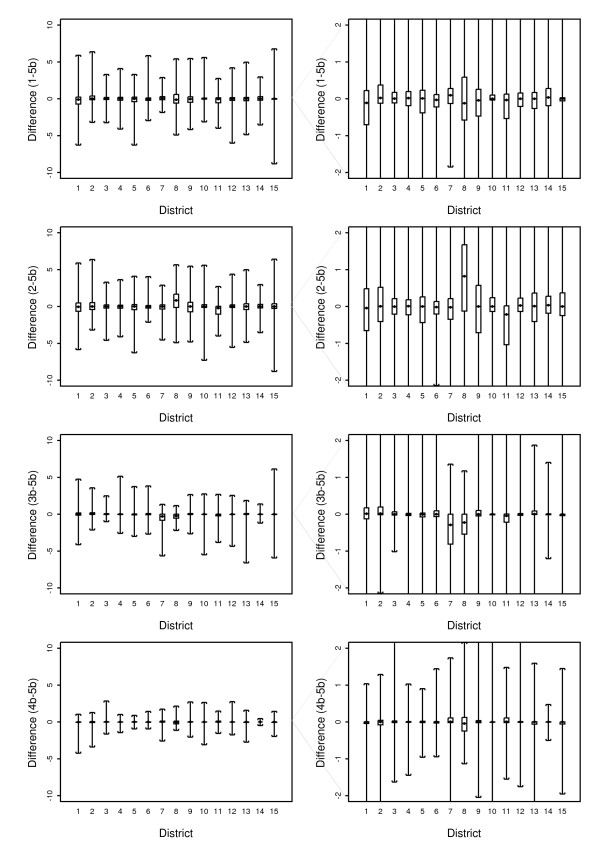
**Box plots of the temperature differences for the 5 Options by climatic zone**. The left plot shows the range (whiskers) and the right hand plot shows the median, first quartile and the third quartile (box).

The temperature differences were also grouped by region and are shown by box-plots in figure 12. These do not vary as much between regions and in all regions 50% of daily differences between Option 3b and 5b are less than plus or minus 5 degrees Celsius. In Option 4b, 50% of the daily differences are within 0.3 degrees different from Option 5b. Districts 7 and 8 stand out in this comparison as well showing a larger difference between Options 3b and 5b compared with the other districts.

### Temporal differences

To see if there was variation in the daily differences from Option 5b during the year, the differences were also grouped by month. There were greater precipitation differences for both Options 3b and 4b in February 2002, a month with high rainfall in some parts of NSW, increasing the likelihood of greater differences.

The differences for daily temperatures grouped by month showed that the daily differences for Option 3b in the winter months June, July and August were more strongly negative than those in Option 5b.

## Discussion

The primary focus of this work is a comparison of options for calculation of weather exposure measures for health analysis of small area populations. The exposures were average temperature and rainfall, although other measures could be similarly compared including humidity, ultraviolet radiation, air pollution and other environmental exposures. The criteria used to assess the different options were: conceptually sound; computer time required; low variation across methods; and completeness of values at the daily POA level. The population weighted average of inverse distance weighted averages (Option 5) fulfilled these criteria best. The other geographic and population weighting methods performed similarly, and were quite close to the Option 5 estimates in most regions. The use of data from weather stations internal to the area or using neighbour allocation methods based on proximity polygons performed poorly. This was because the density of the monitoring stations is very low resulting in dependence on only a few observations to calculate values.

A possible limitation of Option 5 is that the population used to describe the fine resolution distributions were based on the August 2001 census enumeration counts. This single estimate may not be an accurate representation of the population at other times. If the data are available then the distribution of population at specific times could be taken into account in the calculations. As this study calculates weather estimates shortly after the census this issue will not greatly affect the application presented here. As the census is based on residence, the population distribution does not take account of frequent movement of people such as daily travel to other areas for work, which may differ by population. In the absence of such data describing movement of people, the census currently represents the best available data on population distribution.

The nearest neighbour method (using proximity polygons) allocates less monitoring stations to each POA and thus limits access to regional information and may give unrepresentative estimates. This also causes these methods to be susceptible to large gaps in the series. The problem of missing data in Options 1 and 2 could be resolved in a number of ways by imputation. However problems associated with having less monitoring station observations per POA cannot be easily dealt with in this approach.

The inverse distance weighting approaches incorporate information from many more stations. For this reason they are less susceptible to the gaps found in Options 1 and 2. However when no stations are close then far off stations are given full weight. We set the limit at 50 km. As some POA estimates were derived from stations almost this far from their centroids these values may be untrustworthy. A tighter search radius would reduce this, but would increase the number of missing values, while a larger radius would incorporate more values but potentially more unreliable data. Sensitivity analyses could be done to study the effect of the different cut-off levels.

Option 5b was based on localised population weighting. This gives higher estimates at greater rainfall magnitude than any of the other methods using non-squared distance weighting. In some coastal areas of Australia there is highly localised intense rainfall, which is the probable cause of this effect.

The inverse-squared-distance-weighting options (3b and 4b) decreased the influence of stations at a greater distance and gave more similar results to Option 5b.

## Conclusion

Daily temperature and rainfall estimates calculated by using data from internal sites or nearest neighbours (proximity polygon) methods give poor representations of local area weather patterns for health studies based on daily data. The weighting approaches using weather stations less than 50 kilometres from area centroids were considerably better in this regard and the majority of daily differences across the options were small. The extent of the differences depended to some extent on the climatology of the location of the spatial unit and the time of the year. For studies of human health in the Australian context the distance to a regional geographic centroid is not as precise as a population weighted centroid, as large areas of uninhabited land (and the weather of these areas) may not provide relevant information about weather exposures. The population weighted average of sub-unit inverse distance weighted estimates is the most conceptually appealing method applied here. However, it is more computationally intensive than simpler population weighted centroid estimates and there is little difference in daily average temperature and rainfall estimates.

## Methods

### Hardware and software

Options 1 to 4 were calculated on a desktop PC. Option 5 was performed on a Structured Query Language (SQL) server. GIS operations used ArcGIS 9.1 [40]. Microsoft Access was used to join the concordance table of POA-to-monitoring station proximity polygons to the daily observations, and averaged these whilst grouping by POA code and date in Options 1 and 2. Options 3 and 4 used "joinby" and "collapse" commands in STATA 8 [41] to join the distance weights with the daily observations, and Option 5 used the SQL server.

### Meteorological data

Individual station files of daily meteorological data for 1990–2005 were parsed for integration in MS Access databases using visual basic code written by Melissa Goodwin at the National Centre for Epidemiology and Population Health.

### Postcode/postal area populations and concordance

The CD populations from the 2001 census were obtained from the ABS [36]. These data were enumeration counts rather than area of usual residence which cost more.

Some postcodes don't exist as POA and for these the locality names were found using the online postcode finder from the electronic telephone directory [42]. These locality names were georeferenced using the online Geoscience Australia Place Name Finder [43] or the ABS 'Urban Centres and Localities' spatial boundaries (also CD aggregates from the ABS). These locations were then overlaid and intersected with the POA boundaries and given this code instead of their real postcode.

Multipart POA were assessed by first using the ArcGIS multipart to single-part tool (features toolbox) and then counting the number of parts per feature (using the frequency tool).

### Internal stations

Internal stations were found using the intersect tool in the ArcGIS Spatial Analyst extension. This information was joined to the meteorological data using Microsoft Access.

### Nearest neighbour

Nearest neighbour concordances were calculated by first creating proximity polygons of the appropriate stations (using the coverage tools), then overlaying and intersecting these with POA (using Spatial Analyst tools in ArcGIS).

### Distance

Centroids were calculated using the Visual Basic for Applications script from the ArcGIS help menu. Then distances were calculated using the coverage toolbox "point-distance" tool. The projection was set to Albers South Asia Conic (metres) projection. This is necessary to avoid the distortion of length inherent with other cartographic projections [44].

## Competing interests

The author(s) declare that they have no competing interests.

## Authors' contributions

IH carried out the GIS analysis and drafted the manuscript. GH conceived the study, conducted the health analysis and helped to draft the manuscript. KD provided theoretical and conceptual guidance and helped to draft the manuscript. All authors read and approved the final manuscript.
